# Case of Disease in the Larynx Mistaken for Stricture of the Œsophagus

**Published:** 1822-09

**Authors:** John Shaw

**Affiliations:** Lecturer on Anatomy and Surgery.


THE LONDON
### Medical and Physical Journal.

3 OF VOL. XLVIII.] SEPTEMBER, 1822. [NO. 283.

For many fortunate discoveries in medicine, and for the detection of numerous errors, the world is indebted to the rapid circulation of Monthly Journals; and there never existed any work, to which the Faculty, in Europe and America, were under deeper obligations, than to the Medical and Physical Journal of London, now forming a long, but an invaluable, series.—RUSH.

### ORIGINAL COMMUNICATIONS, SELECT OBSERVATIONS, &c.

ART. I.—*Case of Disease in the Larynx mistaken for Stricture of the Œsophagus.* Communicated by JOHN SHAW, Lecturer on Anatomy and Surgery.

I WAS lately requested by a physician to examine the body of a gentleman who had died of a disease in the throat, and on the nature of which much difference of opinion had existed. The following is a slight outline of the history of the case.

The first symptoms were those of pain in the throat with expectoration of blood; after this had continued for some time, the patient found great difficulty in swallowing; he had also a copious puriform expectoration. This combination of symptoms led the physician in attendance to suspect that there was stricture of the œsophagus, and probably disease of the lungs. As the stricture of the œsophagus was supposed to be the cause of the most distressing symptoms, an eminent surgeon was consulted, who, taking the same view of the case, introduced bougies into the œsophagus, with the intention of relieving the stricture. But this attempt was not followed by any advantage. A third physician was now called in; and he gave it as his opinion that the difficulty of swallowing was not caused by a stricture, but by spasm of the muscles of the pharynx, which was produced immediately on the food coming in contact with the diseased and irritable larynx; and the puriform expectoration he considered to be only a secretion from ulcers in the larynx, as there was no other symptom which indicated disease of the lungs. With this view of the case, he prescribed opiates, which had an immediate effect in obviating the difficulty of swallowing.

The question of the existence of a permanent stricture in the œsophagus was now decided in the negative; but, in opposition to the opinion that the disease was confined to the larynx, it was stated that the patient expectorated so large a quantity of matter, that there could be no doubt of the lungs being diseased. The patient gradually sunk, and at last died apparently from excessive irritation of the larynx, with, however, occasionally such marked symptoms of stricture of the œsophagus, that the physician who had asserted that there was no stricture was very anxious that the question should be determined by an examination after death.

On the lid of the coffin being raised, the physician was not a little surprised to find a large bloated and putrid body, instead of the thin emaciated figure of his late patient. This was the more inexplicable as the patient had not been dead more than forty-eight hours, and the weather had been particularly cool. To my eye, the body had much more the appearance of one who had died suddenly of some very acute disease, than of that of a patient who, as I was told, after having been reduced almost to a skeleton, had at last sunk under the irritation of a chronic disease of the throat.

On touching the body, I found it to be emphysematous, and, on cutting through the skin, a quantity of very offensive gas escaped.

Since the appearance of a body a short time after dissolution is sometimes considered indicative of the cause of death, it becomes important to inquire into the reason of the putrefaction having advanced so rapidly in this instance; particularly as it is generally a long time before the bodies of those who die of a lingering disease become putrid.

To account for the putrid state of this body, I at the time hazarded an opinion, which, though certainly speculative, and perhaps erroneous, I will venture to repeat, in the hopes of exciting the attention of others to the question: but this I shall reserve until I have given an account of the appearances of disease which were presented on dissection.

The tonsils and the back part of the tongue were found to be slightly eroded, as if from a fretting inflammation; the surface of the epiglottis had precisely the same eroded appearance as that observed in old cases of cynanche laryngea, and with a character very similar to that of several of the preparations preserved in Mr. Charles Bell's collection.

The sacculi of the larynx were ulcerated, and the edges of the cordæ vocales were thickened and eroded. The membrane of the larynx was inflamed and thickened; but the marks of inflammation became gradually less towards the bronchii. The lungs were found distended with air, which was with some difficulty pressed out, as there was a slight degree of emphysema in their substance. They were also in some parts adherent to the ribs; and, on cutting into them, a considerable number of small tumors were found, the appearance of which corresponded to the description given by the French pathologists of the millet-seed tumor. However, upon the whole, the lungs were not much affected: indeed, they were in a much better state than is often found in those who die without having ever had any symptom which might have led us to suspect that the lungs were affected.

The upper part of the pharynx was ulcerated, but only in a degree corresponding to the eroded or fretted state of the tonsils. Here I may observe, that this irritable state of the tonsil is often mistaken for ulceration, and particularly towards the end of a course of mercury given for primary venereal symptoms; for, about this time, the ducts or follicles of the glands enlarge, their edges become red and fretted, and the secretion of the gland is generally viscid and adhesive. In consequence of this combination of circumstances, the tonsil acquires so much of the character of venereal ulceration, that there is danger of a patient being put under a second and severer course of mercury. I have known instances of this. To distinguish between this irritable state of the tonsil and one affected by a venereal ulcer, we have only to press upon the side of the gland with the tip of the finger, or with a probe; for by this we shall push out the viscid secretion which is lying in the enlarged follicle, and then the supposed ulcer will appear clean.

The œsophagus was now carefully examined, but there was not the slightest obstruction found in it. I could pass two fingers through the narrowest part of the tube.

It is unnecessary to make any observations on the importance of distinguishing between the difficulty of swallowing occasioned by irritation in the larynx, and that by a stricture of the œsophagus; or on deciding whether the expectoration of pus is from ulcers in the larynx, or from the lungs.

I shall not, however, enter upon the subject further than to make a few observations with the view of directing the attention of others to the question.

Examples of cases where there was difficulty of swallowing in consequence of disease in the larynx, and very similar to the one just detailed, may be found in the first Number of the "Surgical Observations," by Mr. Charles Bell; and I have no doubt that such cases have been seen by most practitioners. It also must have been observed, when there is disease high up in the œsophagus or in the pharynx, that there is an occasional difficulty of breathing, and particularly when the parts are in an excited state. There can, I think, be little doubt, when disease of the one organ produces a corresponding state of the other, that it is in consequence of the intimate connexion which there is between their nerves.

Within these few days, I have had an opportunity of observing, in a case of hydrophobia, a most horrible example of the combination of the actions of the pharynx and larynx. Indeed, the circumstance of the symptoms in hydrophobia being in every case nearly the same, although, upon dissection, the inflammation is sometimes found in the œsophagus and sometimes in the larynx, is a striking proof of the truth of the opinion, that when one of these organs is irritated there will be a corresponding state of the other.

To prove farther, that when two organs are supplied with the same set of nerves, and when the proper play of these actions are dependent on each other, that an excited state of the one will produce a corresponding condition of the other, we might adduce examples from the morbid state of other organs. Thus, how often is irritation of the rectum mistaken for stricture in the urethra, or a slight irritation in the urethra for a disease of the rectum? It is also worthy of remark, that the circumstance of the irritation of the one being relieved by soothing applications to the other has given rise to many mistakes: for it is owing to this that one practitioner will insist that all uneasy feelings about the perineum depend on the state of the rectum, while another will be as positive that they have their origin in a disease of the urethra.

The question which was agitated, during the patient's lifetime, as to the source of the matter which was expectorated, is very important. In a case where the symptoms of suffocation called for the operation of tracheotomy, it was not performed, in consequence of so large a quantity of matter being expectorated as to induce the physician to suppose that the lungs were diseased, and that, consequently, the operation would be unavailing. I had an opportunity of examining the body of this patient: I found the disease to be entirely confined to the larynx, the lungs being free from tubercle or ulceration. They were, however, in a state of congestion, and their substance was slightly emphysematous; which I suspect was, as in the case here discussed, a consequence of the laboured respiration caused by the irritated and convulsed state of the muscles of the larynx.

This view of the case naturally brings me back to a consideration of the circumstance which was mentioned in the first part of the paper,—viz. the putrid state of the body; for I suspect that it was in some degree owing to the difficulty of respiration. The opinion I offered during the dissection was, that the disease had been of such a character as to have produced a difficulty or obstruction to the exit of the inspired air; and that, in consequence of this, an emphysema, similar to what occasionally occurs in women during labour, had been produced immediately before death; and that the inspired air, lodging upon the cellular membrane, had been the cause of the rapid decomposition of the body.

This opinion I formed, not from any recollection of the appearance of the bodies of those who have died from diseases of the throat corresponding with the state of this one, but from the observations which I had made on the condition of those who have died of suffocation. However, as the analogy is liable to objection, I shall state a few of the circumstances which induced me to form it.

I had frequently noticed, that when an animal was destroyed by blowing air into its veins (from the lungs,) that it became putrid much more quickly than one which had been bled to death, or had been killed by a blow; and I have further observed, that the parts in the neighbourhood of the heart, where the air has lodged, were the first that became putrid. I have also noticed, that when emphysema has taken place in an executed criminal, (which it very often does in the struggles of suffocation,) that the part of the body where the air collects is sooner destroyed than any other portion.

From having observed the same phenomenon several times, I have been naturally led to suspect that the putrefaction was caused by the chemical action of the respired air upon the dead cellular membrane. I cannot offer any distinct experiments to show that there is a difference in the effect of respired and atmospheric air upon dead animal matter, (that neither of the airs having any bad effect upon living matter, is proved by those cases of emphysema which are caused by fracture of the ribs;) but we have occasional opportunities of observing their effect on bodies in the dissecting room:—as, for example: in the middle of winter, the bodies of two very powerful young men, who had been drowned, were brought in. Though they had been only a very short time in the water, they became, in the course of twenty-four hours, black and puffed up, and particularly about the chest and neck. On raising part of the skin, a quantity of very offensive gas escaped. Conceiving that the cause of the rapid decomposition into which these bodies were going was the lodgment of the respired air, I removed a quantity of the skin, so as to allow of a free exit of the air. We then observed that those parts of the body from which the skin was removed, and the gas pressed out, and which were of course exposed to the action of the atmospheric air, did not go so rapidly into complete putrefaction as where the gas was confined by the skin. This question may, perhaps, be considered of sufficient importance to induce some to institute a series of experiments to show the different effects which the variou gases have upon dead animal matter. The observations which I have made are of too limited a nature to enable me to proceed further in it at present.*

* It may be supposed that the emphysematous state of these bodies was produced, not by the expulsion of air from the lungs, as in the executed criminal, but by their immersion in water.

In objection to this I would state, that the bodies of these men were only a very short time in the water, and that a dead body will not become emphysematous in consequence of lying in water until after a considerable time; indeed, not for weeks during the winter. We must therefore suppose that some other cause is operating, since there is so great a difference in the state of the body of a person who has been drowned, and that of one which has been immersed in water after death. I suspect that the emphysema is caused by a certain quantity of the air which has been inspired during the occasional rising of the drowning person to the surface of the water, having escaped through a rupture produced in the bronchia or trachea in the convulsive attempts to breathe.

